# A rapid realist review on leadership and career advancement interventions for women in healthcare

**DOI:** 10.1186/s12913-024-11348-7

**Published:** 2024-07-28

**Authors:** Doreen Mucheru, Eilish McAuliffe, Anosisye Kesale, Brynne Gilmore

**Affiliations:** 1 UCD School of Nursing, Midwifery and Health Systems, University College Dublin, Dublin, Ireland; 2 Mzumbe University, Morogoro, Tanzania

**Keywords:** Leadership, Women, Healthcare, Career advancement, Interventions, Programmes, Gender equity

## Abstract

Women represent 70% of the global health workforce but only occupy 25% of health and social care leadership positions. Gender-based stereotypes, discrimination, family responsibilities, and self-perceived deficiencies in efficacy and confidence inhibit the seniority and leadership of women. The leadership inequality is often compounded by the intersection of race and socio-economic identities. Resolving gender inequalities in healthcare leadership brings women’s expertise to healthcare decision making, which can lead to equity of healthcare access and improve healthcare services. With the aim of enhancing women’s advancement to leadership positions, a rapid realist review (RRR) was conducted to identify the leadership and career advancement interventions that work for women in healthcare, why these interventions are effective, for whom they are effective, and within which contexts these interventions work. A RRR ultimately articulates this knowledge through a theory describing an intervention’s generative causation. The Realist and Meta-narrative Evidence Syntheses: Evolving Standards (RAMESES) for conducting a realist synthesis guided the methodology. Preliminary theories on leadership and career advancement interventions for women in healthcare were constructed based on an appraisal of key reviews and consultation with an expert panel, which guided the systematic searching and initial theory refinement. Following the literature search, 22 studies met inclusion criteria and underwent data extraction. The review process and consultation with the expert panel yielded nine final programme theories. Theories on programmes which enhanced leadership outcomes among women in health services or professional associations centred on organisational and management involvement; mentorship of women; delivering leadership education; and development of key leadership skills. The success of these strategies was facilitated by accommodating programme environments, adequacy and relevance of support provided and programme accessibility. The relationship between underlying intervention entities, stakeholder responses, contexts and leadership outcomes, provides a basis for underpinning the design for leadership and career advancement interventions for women in healthcare.

**Supplementary Information:**

The online version contains supplementary material available at 10.1186/s12913-024-11348-7.

## Background

“Healthcare is delivered by women but led by men”— women are grossly underrepresented in leadership relative to their participation in the health workforce [1]. They represent 70% of the global healthcare workforce and 59% of medical, biomedical and health science degree holders [1, 2]. Nonetheless, women only occupy 25% of health and social care leadership positions [3]. Moreover, women typically assume lower-status and lower-paying jobs in health and social care [1]. Gender-based stereotypes and discrimination inhibit the leadership and seniority of women, which may be compounded by intersection with race and socio-economic identities [1]. These inequalities reduce career satisfaction, morale and lifetime income among women [1].

Gender inequality is a pressing human right and socioeconomic issue with downstream outcomes such as poorer health among women [1, 3]. Addressing gender inequalities may increase the number of role models and mentors for women [1, 3]. Women role models and mentors influence career advancement as they can advise on balancing career and family and counsel on career progression opportunities [4]. Instituting women leaders may also improve the attitude towards their leadership, and enhance identification of leadership with women peers [5]. The presence of women leaders creates a platform for greater emphasis on issues that impact women and girls such as sexual and reproductive health [1, 3]. Projections from the World Health Organization (WHO) Gender Equity Hub of the Global Health Workforce Network indicate that addressing gender inequalities will accelerate the attainment of Universal Health Coverage (UHC) and Sustainable Development Goal (SDG) targets [1]. Progression towards gender parity will also fuel economic growth, and has been estimated as translating to a global gross domestic product increase of US$12 trillion within a decade [6]. More broadly, increasing opportunity for the participation of women is an investment towards organisational success, national prosperity, and quality of life [2].

Extant literature in the field of gender and leadership primarily focuses on barriers that hinder the uptake of leadership positions among women, with a smaller subset focusing on potentially effective strategies to advance women’s leadership [2, 7–13]. Barriers that inhibit women’s leadership include decreased capacity owing to career disruption and family responsibilities, unfavourable credibility assumptions, and perceived deficiencies in self-efficacy and confidence [2]. The literature on interventions which advance women’s leadership highlights that these interventions engender career advancement among women, promote knowledge and skill acquisition, enhance wellbeing and morale, encourage staff retention, augment remuneration, and progress organisational culture and practices towards gender equity [2, 7–13].

Systematic reviews and literature summaries on this topic exhibit certain oversights, such as insufficient description of primary studies, inadequate focus on research in the healthcare context, and lack of a standardised approach in evaluating the research [2, 7–13]. Further limitations are that many studies focus on single intervention strategies (i.e., mentoring, networking), insufficiently describe intervention components, use variable terminology to define similar intervention concepts, have poor methodological rigour, display wide heterogeneity in outcomes measured, and exhibit missing process evaluations [2, 7–13]. Moreover, there is little to no research in low and middle-income contexts (LMICs) and a paucity of system-based or culture focused interventions [2, 7–13].

With these limitations in mind, evidence synthesis on leadership and career advancement interventions for women needs to examine why certain programmes are more or less likely to work in certain ways, for specific people and in particular circumstances through contributing to theory [14]. A theory is transferable and applicable across a range of circumstances [14]. The links between interventions, contexts in which these interventions are implemented, participant responses to interventions, and outcomes of interventions are not evident in the existing literature [14]. This gap is consequential to the design of reviews that do not provide insight on how programmes work and how this may change based on settings or circumstances of key actors [14].

Establishing evidence-based programme theories on women’s leadership and career advancement interventions, especially, for healthcare workers is relevant for global and national policy [15, 16]. Gender equality is an objective and driver for attaining the United Nation’s Sustainable Development Goals [15]. Accordingly, the aim of our research is to identify what leadership and career advancement interventions work for women in healthcare, why exactly these interventions are effective, for whom specifically they are effective, and the contexts of operation. Although not part of the current research, review findings will partially inform the development of a leadership and career advancement intervention for women within the Tanzanian healthcare setting.

## Methods

### Design

A rapid realist review (RRR) was conducted to answer the research question, “What leadership and career advancement interventions work for women in healthcare, why exactly are these interventions effective, for whom specifically are they effective, and in what contexts?”. The RRR is described as ‘rapid’ because the search is expedited by reducing the number of databases searched and decreasing iterations within the synthesis [14]. A RRR ultimately provides a theory that indicates what programmes are likely to work, for a specific target group and under a particular set of circumstances [14]. Uncovering theories can improve the effectiveness, acceptability, transferability, and sustainability of programmes [17]. A key advantage of the RRR methodology is that it is responsive to local policy needs, and results are utility-focused [14]. The RRR approach is appropriate because this methodology effectively dissects complex programmes by scrutinising what components work, for whom exactly, under what circumstances, and why this is the case [18].

A RRR uncovers the generative causation of a phenomenon, which is expressed as a context mechanism and outcome configuration (CMOc) [18]. Context (C) can be defined as environments and pre-existing conditions within which interventions are introduced [18]. Mechanisms (M) are underlying intervention entities, processes, and structures that operate to generate outcomes (O) [18]. Mechanisms are a combination of resources (R1) introduced and the stakeholder’s reasoning (R2) in response to the resource [18]. Outcomes are consequential to mechanisms acting in contexts [18].

The RRR aims to uncover generative causation – the underlying causal processes that generate an outcome, either intended or unintended [14]. Generative causation which is expressed as a CMOc, describes the mechanisms triggered within specific contexts and outcomes this interaction generates [18]. Elucidating the link between these components uncovers the programme theory, which explains how the interventions work [18]. This theory is tested and continuously refined throughout the RRR [14]. The theory generated is not prescriptive but takes an explanatory approach [14].

**Fig. 1:**
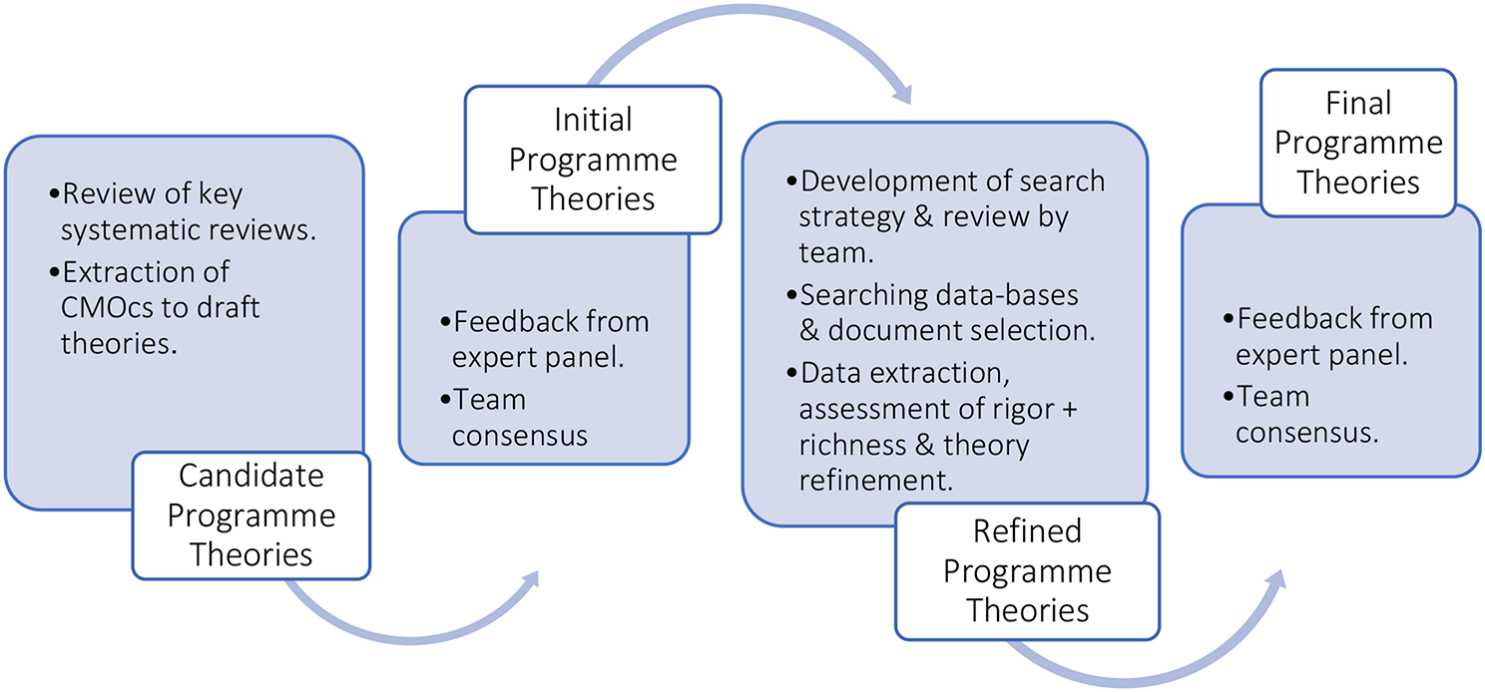
Key steps in the rapid realist review [14, 20]

This RRR was conducted between December 2022 and November 2023. The review design comprised of the phases: identifying existing theories, literature searching, document selection, data extraction, appraisal for richness and rigour, data synthesis, validation and programme theory refinement, and dissemination of findings [19]. Figure 1 details the steps taken within this RRR.

### Expert panel

Aligned to RRR guidance and best practice, the expert panel was convened to help inform the research direction, ensure the relevance of the review to low and middle-income settings and more particularly to the Tanzanian healthcare context, and contextualise findings to ensure that the findings have real-world input. The expert panel consisted of 16 people. Tanzanian members of the expert panel were recruited during a visit to Tanzania, where a presentation was made to government officials and healthcare staff on the necessity of a contextually befitting leadership and career advancement intervention. Non-Tanzanian members of the expert panel were recruited through existing networks and by identifying key individuals involved in policy, programme implementation and research who could support this work.

Members of the expert panel included:

I. Doctors, nurses and allied health professionals (nutrition and social welfare officers, diagnostic health service employees) from Tanzania – 8 members.
II. International experts in gender, health systems and global health — 8 members.

The expert panel were consulted during the defining stages of the review, including the drafting of initial programme theories and refinement of final programme theories. Meetings were thus held during these two critical timepoints. The panel were asked to provide feedback after reviewing theories during in-person and online meetings (Zoom). These meetings were of varying sizes as they were based on the schedules of expert panel members. The expectation was for expert panel members to participate in at least two key meetings lasting no more than 1.5 h. Where expert panel members could not attend the meetings, feedback was solicited via email. Expert panel feedback was implemented after consultation and consensus with the core research team.

### Identifying candidate and initial programme theories

Key systematic reviews on leadership and career advancement among women were identified using the keywords leadership, career, interventions, and women in the Google Scholar search engine. This initial literature scope was not meant to be exhaustive but rather to inform the research disposition. In concordance with this, five key reviews pertinent to the topic under scrutiny were identified and reviewed to identify the candidate programme theories—see Appendix 1 for information on these reviews [2, 7–9, 13].

From these five reviews, three candidate programme theories were constructed. These were then presented to the RRR expert panel and the wider research team, who both provided feedback. The candidate programme theories were then refined into initial programme theories (IPTs) [18, 20]. The resulting IPTs were:

I. Interventions targeting women’s leadership should take a multi-component approach that targets different systems levels and different genders across different sectors. Multicomponent interventions result in the greatest skill development and career advancement for women when they combine individual growth (training, education, mentoring and networking) with wider organisational gender equity strategies (changes in organisational processes or culture) in the context of government and societal strategies.
II. Mentoring with a multidimensional focus (career and other aspects of life) is central to the success of multicomponent interventions targeting skill development and career advancement among women. Mentoring produces the greatest skill development and career advancement among women when the relationship between mentor and mentee is organic/genuine, within a supportive network and the actual mentoring enacted by more experienced colleagues.
III. Leadership development intervention programmes should be structured and supported by wider organisational, societal and government gender equity strategies. Using tools, resources and action plans for implementation and monitoring can support accountability and commitment, and produce measurable outcomes at the organisational level.

### Literature searching

Study search terms were developed from keywords identified in the IPTs. Aligned to RRR methodology where database searches are limited, databases were restricted to CINAHL and Web of Science because the subject matter covered relevant topic areas including nursing and allied health research, healthcare sciences, and social sciences [14]. Search terms addressed the four areas of interest: leadership, interventions, healthcare, and gender. The comprehensive search strategy is detailed in Table 1. Searches were run for studies conducted among adults and published in English between January 2000 until 7th March 2023. The 2000 cut-off date was chosen because this coincided with the release of the United Nations Millennium Development Goals on the promotion of gender equality and the empowerment of women [21]. Members of the expert panel were also contacted twice via email with requests to recommend potentially relevant literature.

**Table 1:** Comprehensive search strategy

Keyword	Leadership	Intervention	Healthcare	Gender	
*BOOLEAN OPERATOR*	AND	AND	*AND*		
*KEY WORD ALTERNATIVES*	Leader* OR “Career Advance*” OR “Professional Development*” OR Mentor*	Interven* OR Training* OR Programme* OR Educat* OR	“Health worker” OR “Health careworker*” OR “Health care worker* OR “Health professional” OR “Health care professional*” OR “Healthcare professional*” OR “Healthcare provider*” OR “Health care provider*” Nurse* OR Midwife* OR Medic* OR Doctor OR Pharmac* OR	Wom? n OR Female* OR	

### Selecting documents

Citations identified during searching were exported into software (Covidence) where duplicates were removed. Search results were initially screened by title and abstract, then by full text based on definitive criteria pertinent to the research topic (see Table 2 for relevance criteria). This screening was completed by DM, and 10% of the studies screened were counter-checked by AK at the title and abstract stage. There were less than 5% of conflicts for studies screened by DM and AK; BG acted as the arbitrator where the two reviewers could not resolve conflicts.

**Table 2:** Relevance criteria for inclusion and exclusion of Papers and documents

Inclusion Criteria	
1.	Interventions delivered to women with any demographic characteristics and in the health sector OR Interventions delivered to both men and women.
2.	Described an individual-focused intervention or an organizational-level intervention or a government-level intervention.
3.	The intervention was designed specifically for advancing women in leadership or the forward momentum of their careers or to advance gender equity.
4.	Outcomes were assessed (either included quantitative and qualitative methods).
5.	If the paper is not an intervention, then it contributes to knowledge and understanding of advancing women in leadership or the forward momentum of their careers in healthcare.
Exclusion Criteria	
1.	Studies that solely focused on the reporting of barriers or enablers related to gender equity broadly, with no interventional element or that did not include at least one outcome related to advancing women in leadership or their careers

### Evaluating richness and rigour

Papers and documents deemed relevant to the research topic were reviewed for richness. Richness assessments were based on the inclusion of sufficient depth to meaningfully contribute to theory building as indicated by having traceable CMOcs; theories of interest were either the initial programme theories or other theories relevant to the topic under scrutiny [22]. Studies were scored 1 point for each CMOc that was evident.

Rigour was applied to assess the methodological conduct of the included papers and documents. Rigour was assessed based on a yes (1) or no (0) dichotomy for the credibility of the source, appropriateness and trustworthiness of methodology used, and plausibility of the information reported [22]. Summary of richness and rigour scores can be found in Appendix 2.

### Data extraction

The first phase of data extraction entailed collating information on the paper’s aims, setting, participants, design, intervention details, findings, and theoretical frameworks and models. After this data had been gathered, the richness and rigour of the papers were determined.

Data pertinent to generative causation including the context, mechanism and outcome of each CMOc was extracted and aggregated. This process entailed reading each paper and extracting information pertaining to the environmental and pre-existing conditions where interventions were introduced which was identified as the context. The details on the resources introduced, stakeholders and their responses to introduction of a resource was extracted and categorised as the mechanism. Finally, the resulting outcomes of the interaction between the introduction of resources and stakeholder responses were also identified. This information was utilised to draft CMOcs for each paper which can be found in Appendix 3.

### Data synthesis

The IPTs were tested and revised in light of the newly emerging data from the CMOcs. Strategies used to test and refine programme theories included [23]:

a) Juxtaposing- contrasting evidence on mechanisms in one source to elaborate outcome patterns in another source.

b) Reconciling- identifying explanations for different outcomes by unveiling contextual differences.

c) Adjudication- clarifying reasons for contradictory study outcomes based on methodological variances.

d) Consolidation- constructing explanations for how and why dissimilar outcomes occur as pertinent to a specific context.

e) Situating- distinguishing which mechanisms were activated in specific contexts.

## Results

**Fig. 2:**
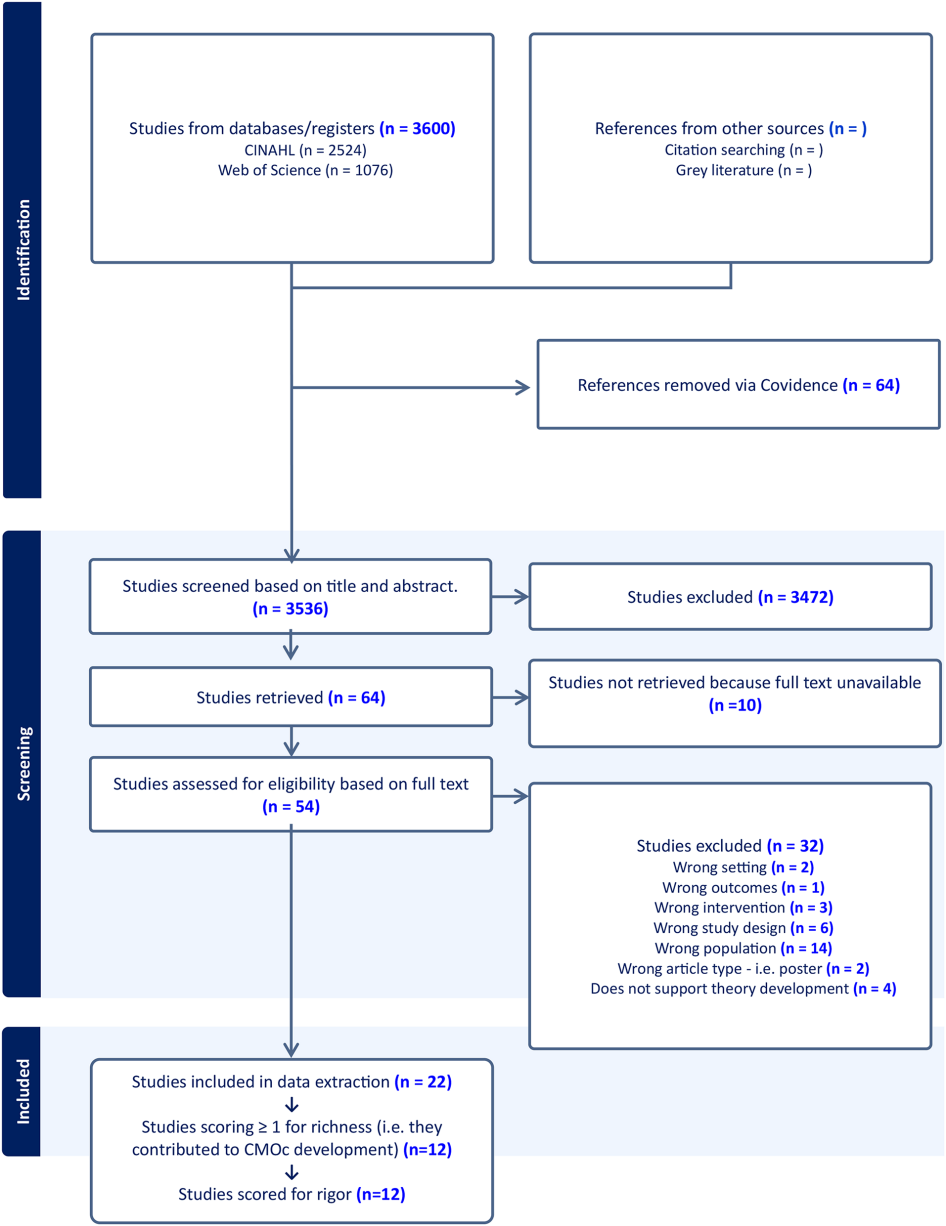
PRISMA flow chart for studies evaluated for the rapid realist review

A total of 3,600 records were retrieved after searching the databases. Sixty-four duplicates were removed, leaving 3,536 papers which were screened at the title and abstract stage, after-which 3,472 were excluded. 64 papers underwent full text screening of which ten were automatically excluded due to the absence of a full text. During full text screening, 32 papers were excluded for various reasons (see Fig. 2), leaving a total of 22 studies that met inclusion criteria and underwent data extraction.

After the first phase of data extraction, only 12 studies were appraised as rich because they contributed to CMOcs, subsequently supporting theory development. These 12 studies were also rated for rigour. Appendix 2 presents a summary of ratings for richness and rigour.

A total of 29 CMOcs were extracted from the included 12 studies. Appendix 3 presents a full list of CMOcs. From these CMOcs, nine demi-regularities or patterns were identified, which supported the refinement of the programme theories into nine final programme theories after consultative meetings with the expert panel.

**Table 3:** Final Programme theories with illustrative examples of their presentation within included studies insert table on the next page here

Theory	Illustrative examples (*Examples should be interpreted with prudence as they represent one facet of the theory, and not all the data points that contributed to theory building)
**Theory 1: Organisational and Management Involvement** In health services or professional associations where leadership and/or mentorship programmes for women healthcare workers (C) aligned with organizational goals and existing roles, were inclusive of all genders, and had buy-in from executive and middle management by management being involved in the programme (M-R1), then this bolstered programme success with participants as it enhanced their leadership skills, knowledge, confidence, and self-efficacy (O). This is because the organisational support encouraged involvement and participation, and obstacles related to the planning and execution of the programme were reduced (M-R2) [24–29]. Further outcomes of these leadership and/or mentorship programmes with organisational alignment and management buy-in (M-R1) were that they improved access to leadership positions for women (O), because having buy-in from management can shift the organisational culture to be more supportive and inclusive (M-R2) [24–29]	D Brown-DeVeaux, K Jean-Louis, K Glassman and J Kunisch [24] in their intervention reported: “The mentorship diversity initiative was launched at 2 NYU Langone Health campuses with the *guidance and sponsorship of the system chief nursing officer (CNO)*. The *project aligned with the organizational goal* and the CNO’s appreciation to diversify the nursing workforce and the senior nursing leadership team. ……Following an *agreement with the chief nursing officer (CNO) to support the diversity initiative*, the *project was introduced to the Executive Nursing Council (ENC)* at Tisch and Brooklyn campuses. Both ENCs acknowledged the need for increased diversity at the senior leadership level and endorsed the pilot mentoring program. *All but 1 mentor were members of ENCs*, and mentors were all-inclusive from multiracial backgrounds. “……The *CNO’s sponsorship*,* vision*,* and collaboration with the executive leadership team were foundational for the program’s success*,* including addressing obstacles in planning and execution*. pg 150-pg154”
**Theory 2: Mentorship Pre-training** In health services or professional associations where mentoring programmes were introduced (C), if mentoring pre-training was delivered to women healthcare workers by facilitators who provided information on the mentor-mentee dynamic (i.e. approaching mentors, how to maintain relationships with mentors) and set expectations regarding mentoring (M-R1), this improved programme outcomes including leadership self-efficacy, desire for a leadership role, and acquisition of transformational and clinical leadership skills (O). This is because mentees were more aligned with the process of mentoring and the potential benefits, and there was more engagement from mentees (M-R2) [24, 30, 31].	F Vatan and AB Temel [31] in their in their intervention reported: “The dyads were given a formal mentoring program, *Mentoring and Application Techniques*,* which included two 4-hour sessions*. This *program was conducted to foster the nurses’ understanding about the formal process of giving and receiving mentoring*. It included information on *how to maintain a mentor-protégé relationship* and *how to enhance mentoring skills* through the use of reflective journals describing their mentoring experience and relationship. Thus, the mentors and protégés were informed about how they learned and how they could enhance their respective styles of learning. At the end of the training program, the participants were given a booklet that included the theoretical information regarding the program. …. The *change in scores before and after the transformational leadership inventory* (TLI) formal mentoring program was expected due to the *Mentoring and Application Techniques education at the beginning of the study and the application of the formal mentoring program*. However, in *addition to the effects of the education and mentoring program*,* the experiences during the study as well as the development of interpersonal relationships also brought changes in scores after TLI*. pg 245–248”
**Theory 3: Supportive Mentors** In health services or professional associations (C) where mentorship was delivered to women healthcare workers and more senior members helped to identify and develop leadership competencies (M-R1), this improved mentees’ leadership, self-efficacy, desire for leadership roles, ease of navigating opportunities, transformational leadership skills, and fulfilment of leadership duties (O) because the support of a seasoned colleague provided confidence to the mentees and facilitated access to opportunities (M-R2) [24, 25, 27, 30, 32, 33].	AA Hewitt, LB Watson, C DeBlaere, F Dispenza, CE Guzmán, G Cadenas, AGTT Tran, J Chain and L Ferdinand [32] in their in their intervention reported: “A participants *work throughout the year to design and implement a service project with the assistance of a mentor*. The *purpose of this project is to assist Leadership Academy (LA) participants in recognizing and developing their leadership potential*. Participants select a mentor—someone with current or past leadership experience in Society of Counselling Psychology (SCP)—whom they feel is able to support them in their chosen project. *With their mentor*,* participants develop and implement a project that aligns with the values and current interests of the SCP*. …………Five of 14 participants in this study discussed the role of good mentorship as a facilitator to leadership development. As previously discussed, mentorship is a core component of the LA. *Those who described mentorship as a support noted that their mentors were “influential in their leadership development” and that having “exposure to mentors” was important*. ………….Role modelling by culturally diverse leaders. *Three participants in this study described how the LA exposed them to role models in the field that shared aspects of their social identities*. One participant stated: “I think *that being part of the LA helped me visualize concretely what a person of color or person of a marginalized group can do in a position of leadership*.” It was not only the presence of culturally diverse role models as mentors or faculty that was empowering; *observing these leaders enact leadership seemed to have the strongest impact*. For example, another participant stated that “[seeing how my mentor] conduct[ed] [herself] during professional meetings was also a great learning experience. pg 994–1008”
**Theory 4: In-put/ Co-creation/ Autonomy during Mentorship** In health services (C) where mentorship was delivered to women healthcare workers, and they were given input into the design of the programme, such as scheduling contact and allowed to develop an authentic relationship with the mentor (M-R1), this contributed to the success of the programme among mentees as evidenced by increased leadership self-efficacy, transformational and clinical leadership skills, and desire for a leadership role among mentees (O) because the participants were able to take ownership of the programme and ensured it represented their needs and availability (M-R2) [24, 30, 31].	SG Leggat, C Balding and D Schiftan [30] in their in their intervention reported: “The mentor pairs were divided into three groups and each group had the support of a skilled facilitator to assist the mentoring process. The programme comprised: -three face-to-face workshops for all mentors and Nurse Practitioner Candidates (NPCs), encompassing training in mentoring skills and action learning, educational sessions on clinical leadership, and opportunities for reflection and feedback -*monthly face-to-face or telephone mentoring meetings organised by the mentor pairs*, using an action learning mode ….These *components enabled the mentor pairs to identify leadership issues and have sufficient time over the 18-month programme for the NPCs to consider and complete actions* discussed as potential solutions to the issues. Using the action learning approach (Leggat et al. 2011, McNamara et al. 2014) the mentors were able to encourage the NPCs to reflect on their actions, incorporating the learning for future issue resolution pg 1578 ……There was *a significant increase in skills reported for those items of the (Leadership Practices Inventory) LPI that are associated with transformational leadership* from the start to the end of the programme for the participating NPCs pg pg 1578 and 1581”
**Theory 5: Leadership Education and Content** In health services where (C) relevant and structured leadership training was delivered by content experts who taught women healthcare workers various leadership and discipline-specific topics (M-R1), this improved leadership among participants, including increased leadership self-efficacy, confidence, knowledge, skills, and desire and acquisition of leadership roles (O) because the knowledge gained was seen as applicable to their role, which inspired engagement and commitment (M-R2) [24–28]. Topics for education can include communication, conflict resolution, the feeling of belonging, leader vs. manager, mentoring vs. coaching, wellness, equity, quality improvement methods, leadership styles, critical inquiry, project management, and transformational leadership [24–28].	EH Kelly, T Miskimen, F Rivera, LE Peterson and ST Hingle [25] in their in their intervention reported: “An 18-month curriculum was developed, which included *3 educational series: wellness*,* equity*,* and leadership* (Table 1). *Wellness Through Equity and Leadership (WEL) Topics* ‘*Gender-based differences in burnout and wellness*,* Building your own wellness plan*,* Common workplace inequities*,* Using advocacy to increase representation of women in leadership roles*,* Leadership styles and critical skills*,* Difficult conversations*,* Providing effective feedback* etc….’The curriculum was delivered via monthly webinars and 4 in-person meetings. Eighteen women physicians were enrolled in the pilot implementation of Wellness Through Equity and Leadership (WEL), 3 from each partner organization (the cohort). At the start of the program, participants drafted a statement of purpose and impact (impact statement). Most participants used their statement as a guide for action throughout their involvement in WEL. At the program’s conclusion, cohort members presented their progress toward achieving their goals …….*Participant responses were nearly unanimous that WEL in-person meetings and webinars were key to program success. This included comments about the high-quality curriculum and the importance of having time away from normal life and routines to focus on learning.* In-person meetings were particularly critical to providing participants time to connect with each other. pg s2-s4”
**Theory 6: Practical Components to Support Learning** In health services or professional associations (C) where women healthcare worker participants got to integrate and practice their didactic learning from the leadership education through the incorporation of practical components, such as action learning sets, experiential learning, implementation of a project or interactive sessions (M-R1) then this enhanced leadership outcomes, such as knowledge, confidence, leadership skills and acquisition of leadership positions (O) because practical components equipped participants with a better grasp of the theoretical knowledge, and they got the opportunity to put their skills into practice (M-R2) [26, 27, 32, 34].	P Bradd, J Travaglia and A Hayen [26] in their in their intervention reported: The Allied Health Leadership Development Program was conducted over a ten-month period in 2014–2015 and included *three all-day workshop sessions followed by five action learning sets (ALSs*). For half of the participants in the program, individual coaching support was also provided. *…….ALSs emphasize the importance of the members of the set devising practical solutions to work-based problems themselves* (Haith, 2012). In the context of the leadership development program, *ALSs were seen as an avenue to help participants work through issues as well as to practically demonstrate the use of reflection and enabling questions so they could use these approaches with the staff they supervised.* *The first four ALS sessions comprised 3-h sessions that started with a 1-h presentation* *on a leadership topic that was then followed by the ALS.* Leadership topics were selected by the program participants and included the topics of quality improvement methods, leadership styles, critical inquiry and project management. *After the leadership* *presentation*,* participants were divided into smaller groups for the ALS. The ALS was* *undertaken over a 90-min period.* ……….Data collection also showed that 57% (*n* = 8 of 14) of program participants attained more senior (promotional) allied health positions following the program, compared with 6% of control group members (*n* = 1 of 16). *This finding suggests that increased leadership confidence enabled some program members to successfully apply for more senior position.*pg 910–919.”
**Theory 7: Hybrid Learning** In health services (C) where leadership programmes were delivered among women healthcare workers via both in person and online platforms (M-R1) then this led to increased leadership knowledge, skills, leadership participation and effective fulfilment of leadership roles (O). This is because providing multiplicity of ways to access the programme offered greater opportunity for engagement which enhanced learning and skill acquisition (M-R2) [25, 27, 33]	S Dyess and R Sherman [27] in their in their intervention reported: “*Sessions occurred in classrooms of the academic partner* for the project and the overall project was coordinated by a nursing faculty member from the academic partner. Each session incorporated both didactic content and experiential learning activities designed to help the novices find solutions to the practice environment challenges they faced and spoke about. Throughout the program numerous local, regional, and national nurse leaders presented content and facilitated discussion in the sessions. Between sessions, *the novices participated in Web-based asynchronous learning activities and discussion boards designed to help them apply the skills they learned*. …….Perceptions of the influence the Novice Nurse Leadership Initiative (NNLI) had on the NNLI participants and the practice setting were shared by the managers and mentors of all NNLI participants within focus groups conducted by a researcher other than the project director. Three broad themes emerged as perceived gains: (1) a more global and systems perspective of nursing, *(2) leadership skills that were developed and demonstrated*,* and (3) improved confidence that positively influenced the practice setting on a number of levels* (pg 316–320).
**Theory 8: Leadership Skills Building** In health services or professional associations (C) where leadership development incorporated the attainment of foundational leadership skills, including negotiation, collaboration, networking, reflection, and goal setting among women healthcare workers (M-R1); then this enhanced leadership outcomes such as self-efficacy, leadership knowledge, skills, desire for a leadership role, attainment of leadership positions, and effective fulfilment of leadership roles (O), because the knowledge gained was seen as applicable to their role which inspired engagement and commitment (M-R2) [24, 25, 28, 33, 34].	M MacPhee, J Skelton-Green, F Bouthillette and N Suryaprakash [33] in their in their intervention reported: “The Nursing Leadership Institute (NLI) I is a year-long programme with four components: (i) a 4-day workshop; (ii) mentoring support from higher level leadership; (iii) *organizational supports to implement leadership projects in the practice environment; and (iv) virtual networking (i.e. online community of practice)* (Authors 2009). Since its inception 5 years ago, over 400 nurse leaders have participated in the NLI. Each session is typically comprised of 35–40 leaders (i.e. a cohort) representing a diversity of healthcare sectors and geographical locations in the province All the interviewees stated that *the programme helped them fulfil their leadership roles and responsibilities.* *Nursing focus was another common theme across all four groups.* A front-line interviewee commented: (comparing the NLI to other programs) …in particular the *contacts with the other leaders who were there to talk about things that were specific to my life or my professional life* and to incorporate what other nurses’ experiences have been and to bring that into my practice and to learn from that. A mid-level interviewee stated: I was able to have *discussions with nurse leaders with similar issues*. I think the *networking with these other nurse leaders was so valuable. I’m still in touch with them—it is reassuring*. pg 161–164”
**Theory 9: Self-tailored Learning** In healthcare settings and professional associations (C ) where leadership education delivered to women healthcare workers was self-tailored (M-R1), this ensured the programme represented their needs and resulted in the enhancement of relevant leadership skills (O) because ownership and motivation for the programme was improved (M-R2) [32, 33, 35]	M Cleary, A Freeman and L Sharrock [35] in their in their intervention reported: “This program (clinical leadership program) provided a *more tailored*,* flexible and accessible learning format than those traditionally available in healthcare settings*. It drew from self-directed learning principles utilising a purpose-designed workbook as a framework for participants to develop leadership skills. The *workbook asked participants to develop learning aims and suggested activities that might facilitate their attainment*. *This style of learning enabled participants to set their own time-table*,* which is essential when the time available for training is limited (Corrigan et al.*,* 2001* ……The *workbook provided an opportunity to creatively design activities and outcomes to achieve individual learning aims* and encouraged the use of evidence-based strategies. Opportunities for innovation were considered important as previous research suggests investment must be put into preparing nurses to be creative in their work (Cook, 2001). Participants were *asked to choose a learning outcome* from the following interest areas: • Professional (e.g., best practice, self-assessment, reflective practice, ethics) • Research, evaluation, and quality (e.g., initiate an activity in a specialty area) • Education (e.g., conferences, role modelling, preceptorship, delivering education) • Clinical care (e.g., quality of care, groups, challenging behaviours) • Management/administration (e.g., policies, committees, delegation) • Consultation and liaison (e.g., communication, problem solving, conflict resolution)” …….In addition to the written evidence produced by participants, *other measurable benefits of participating in the program included clinical leadership and succession planning workshops; papers prepared for conferences and publication; attendance at seminars*,* conferences*,* and symposia; and in-service education attended to complement project work*,* for example*,* writing for publication*. ….*On the The Leadership Subscale (LS)*,* 5 of the 6 items had increased*. pg 831–835”

## Discussion

This RRR describes the robust and iterative process of developing programme theories on leadership and career advancement interventions for women in healthcare. By applying the RRR methodology, literature on the topic was appraised and the twenty nine CMOcs which were extracted pointed to the relationship between the programme components, participant responses, programme settings, and resulting outcomes [17]. Demi regularities or patterns were identified within the CMOcs, which led to the construction of 9 programme theories [17].

It was evident that programmes which applied leadership education, training and mentorship for healthcare workers who were women, and were undergirded by management buy-in, inclusion of all genders, alignment with organisational goals and workplace roles, were linked to superior outcomes. Involvement of multiple parties within the organisation and considering organisational dynamics corroborates systems theory which conveys the significance of a wholistic approach [36]. Some organisational entities support, and influence others as is the case with management and other organisational members [36]. It is therefore unsurprising that the women within the health services and professional associations demonstrated improvements in leadership skills, knowledge, confidence, along with greater access to leadership positions [24–29]. Management buy-in such as their endorsement, allocation of resources, and provision of advice regarding the best course of action, minimised planning and execution obstacles [24, 27]. On occasion, management’s endorsement was through integration of the programme to the employment role and pay, which inspired added participant commitment [24, 27].

Obtaining management buy-in provides a level of accountability from the organisation which may motivate women to participate because they view the programme positively [24]. Management’s involvement through nomination of prospective participants potentially had a validating effect on the women [25]. Managers and supervisors at work are powerful agents for demonstrating organisational support, thus their role is indispensable in programmes seeking to establish organisational backing [37].

Mentoring emerged as a core constituent of leadership and career development programmes for women in health services or professional associations [24, 25, 27, 30, 32, 33]. Pre-training on how to approach and relate with mentors seemed to contribute towards leadership skills and self-efficacy [24, 30, 31]. The mentorship enactment theory proposes that proactive communication strategies are essential for initiating, developing, maintaining and repairing mentorship relationships [38]. This pre-training likely aligned mentees and mentors to the process of mentoring, enabling them to reap the maximum benefits because it boosted their engagement and effectiveness [24, 30, 31]. The mentor-mentee dynamic is characterised by an unequal power dynamic between the duo, and some preparatory skills may be required by both parties [38].

The actual mentoring was enacted by senior or more experienced staff who role modelled, provided a sounding board for ideas and assisted mentees with identifying and developing organisationally relevant leadership competencies [27, 32, 33]. It was articulated in some studies that mentors were from different organisations or units thus were not their line managers, and the benefit of this approach is minimised conflict of interest between the mentoring pairs which may contribute constructively towards outcomes [24, 30]. This type of guidance also provided new opportunities for mentees via the network connection of mentors and guided them in the navigation of new opportunities [25]. Benefits of mentorship extended beyond the mentee, and sometimes augmented the mentor’s leadership abilities due to novelty of the role [27].

It was also apparent that providing mentees and mentors with latitude over some aspects of the mentoring boosted leadership outcomes [24]. Two of the interventions indicated that mentorship dyads met monthly however this approach was not consistent across all other studies applying mentorship [24, 30]. Decisions on frequency and scheduling of mentoring meetings, the specific focus areas of the mentoring sessions, overall expectations, and goals was often left to the discretion of the participating dyad after providing general programmatic guidelines on the mentorship process and anticipated outcomes [31]. The self-determination theory posits that humans have a need for autonomy, competence, connection and belonging [39]. When these desires are satisfied, individuals are more likely to be motivated, engaged and successful [39]. Giving mentees and mentors some autonomy may give them a sense of programmatic ownership, which may contribute positively to programme engagement and ultimately towards participant leadership competency.

Leadership education and training was the most notable focus of the interventions [24–28]. This was characterised by leadership and discipline-specific education delivered by content experts which led to leadership self-efficacy, confidence, knowledge, skills, desire for leadership and acquisition of leadership [24–28]. Learning was time-tabled, structured, and assumed majority of programmatic time and resources [24–28]. Delivery of leadership education is congruent with the behavioural leadership theory which asserts that the traits that distinguish leaders can be learnt [40]. Focal areas for the education included transformational leadership, communication, conflict resolution, leadership styles, project management, differences between leading and managing, belonging, quality improvement, wellness and equity [24–28]. Broadly, the leadership topics delivered are relevant to healthcare leadership and perpetuate productivity and growth, which may have inspired additional engagement and commitment [40]. Moreover, delivering this content via in-person and online platforms reinforced positive outcomes because the multiplicity of learning avenues improved accessibility and engagement [41].

This didactic leadership education was typically coupled with practical components, such as action learning sets, experiential learning, implementation of a project and interactive sessions [26, 27, 32, 34]. Although these elements differ quite significantly in execution, they afford an opportunity and context for participants to implement lessons from the education sessions which fortifies leadership knowledge [26, 27, 32, 34]. This is the key message conveyed in the experiential learning theory which postulates that practical experiences facilitate knowledge retention and deeper grasp of ideas [42]. This mode of training is akin to learning by doing where direct instruction is coupled with practical training, and this is superior to traditional learning that is primarily theoretical [43].

Acquisition of skills in negotiation, collaboration, networking, reflection and goal setting among the women healthcare workers was resourced in the leadership education and training, which buttressed leadership outcomes [24, 25, 28, 33, 34]. Relevance of these skills to participants’ workplace roles likely evoked engagement and commitment, contributing to favourable leadership outcomes. Leadership literature cites the importance of communication, negotiation, planning, and problem solving, which parallels the skills cited in the current programmes [44]. Relevance of these leadership skills differs based on the level of organisational responsibility, for instance, planning and problem solving are more requisite at advanced leadership levels [44]. Consequently, it is crucial for programmes to impart leadership skills which are relevant to present and proximal roles [44].

Bespoke learning aims, programme activities, leadership styles and schedule of activities were available within the leadership education and training [32, 33, 35]. This approach enhanced leadership because the programme was tailored to the interests and goals of participants which ignited motivation. Tailored education and training gained traction in recent years due to the impact on knowledge, self-efficacy and behaviour change [35, 45]. Proponents also highlight that the specificity of meeting participant and organisational needs is efficient and time sensitive [46]. Consideration of participant needs and preferences favourably impacts leadership and career development programme outcomes among women healthcare workers.

Limitations

There were ostensible limitations associated with the conduct of this review. Firstly, the leadership and career development programme components were often interlinked, and it was not always clear which components led to certain outcomes and the response of key actors within the scope of the intervention. This therefore hampered information that could be meaningfully extracted from the included studies. Additionally, detailed description of intervention methodology was not always given by the authors which rendered some of the included studies obsolete for the purpose of a RRR, which relies on meticulous reporting and appraisal of intervention mechanisms [18]. Furthermore, the interventions which were explored here generated several theories which may elicit more questions for those implementing the theories due to nuances that may emerge, however these may only be plausible to interrogate at each local level. Lastly, none of the reviewed interventions were set in a low- and middle-income context, which raised questions about the relevance of the theories generated to the Tanzanian healthcare context. The authors attempted to counter this drawback by appraising theories generated with expert panel members from the Tanzanian context to ascertain applicability but clearly more research is needed in these contexts.

## Conclusions

This RRR highlights theories which may be effectual in addressing the underrepresentation of women in leadership through applying leadership and career development programmes in health services or professional associations. Following the literature review process and consultation with an expert panel, 9 theories were developed to guide the development of effective programmes which enhanced leadership outcomes pertinent to acquisition of skills, knowledge, confidence, self-efficacy, fulfillment of existing roles, leadership participation, desire for and attainment of new roles. In general, these programmes comprised of leadership education and training, alongside mentoring. Key strategies applied in the delivery of the leadership and mentorship components was alignment with the organization’s direction and involvement of personnel from management; providing mentorship pretraining, allocating mentors and allowing for co-creation during mentorship; delivering general and discipline-specific leadership education; incorporation of practical components to support leadership education; integration of hybrid learning through utility of in-person and online platforms; development of key leadership skills and creating opportunity for self-tailoring within the programme. These strategies were generally successful because of the supportive programmatic environments, adequacy and relevance of support offered and accessibility of the programmes. The finding that none of the reviewed interventions were set in a low- and middle-income countries underlines an opportunity for testing the theories in this context to comprehensively crystalise features that are suitable. The theories presented underline why, how, for whom and the contexts related to the success of leadership and career development programmes for women in healthcare and can be tested and refined further especially as it pertains to long-term outcomes such as gender equality in leadership.

### Electronic supplementary material

Below is the link to the electronic supplementary material.

Supplementary Material 1

## Data Availability

No datasets were generated or analysed during the current study.

## Abbreviations

RRR: Rapid realist review
RAMESES: Realist and Meta-narrative Evidence Syntheses: Evolving Standards
WHO: World Health Organization
UHC: Universal Health Coverage
SDG: Sustainable Development Goal
LMICs: Low- and middle-income contexts
CMOc: Context mechanism and outcome configuration
R1: Resources
R2: Reasoning
