# Supplementary material for: A rapid realist review on leadership and career advancement interventions for women in healthcare
Source: BMC Health Serv Res. 2024 Jul 28;24:856. doi: 10.1186/s12913-024-11348-7 (PMC11285393; doi:10.1186/s12913-024-11348-7)
Supplement: Supplementary file 1 — Supplementary Material 1 [file 12913_2024_11348_MOESM1_ESM.docx]

# Appendix 1: Reviews Informing Candidate Programme Theories

| Author and Publication Year | Title | Review Period | No of Studies Included |
| --- | --- | --- | --- |
| Mousa, Boyle et al. (2021) [1] | Advancing women in healthcare leadership: A systematic review and meta-synthesis of multi-sector evidence on organisational interventions | 2000-2021 | 91 |
| House, Dracup et al. (2021) [2] | Mentoring as an intervention to promote gender equality in academic medicine: a systematic review | Database inception-2019 | 32 |
| Laver, Prichard et al. (2018)[3] | A systematic review of interventions to support the careers of women in academic medicine and other disciplines | PubMed (1966 onwards) and CINAHL  (1981 onwards)  2017. | 18 |
| Alwazzan and Al-Angari (2020) [4] | Women’s leadership in academic medicine: a systematic review of extent, condition and interventions | Database inception-2018 | 42 |
| Lydon, Dowd et al. (2022) [5] | Systematic review of interventions to improve gender equity in graduate medicine | 1999-2020 | 35 |

# Appendix 2: Summary Ratings for Richness and Rigour for Studies Included in Rapid Realist Review

|  | Reference | Richness rating | Rigour Rating |
| --- | --- | --- | --- |
| 1 | Brown-DeVeaux et al., 2021 [6] | 3 | 3 |
| 2 | Chang et al., 2019 [7] | 0 | - |
| 3 | Kelly et al., 2021 [8] | 3 | 3 |
| 4 | Bradd et al., 2018 [9] | 3 | 3 |
| 5 | Spiva et al., 2020 [10] | 0 | - |
| 6 | Dyess et al., 2011 [11] | 3 | 3 |
| 7 | Leggat et al., 2015 [12] | 3 | 3 |
| 8 | Vatan et al., 2016 [13] | 2 | 3 |
| 9 | Al-Hussami et al., 2017 [14] | 0 | - |
| 10 | Clark et al., 2015 [15] | 0 | - |
| 11 | Cleary et al., 2005 [16] | 2 | 3 |
| 12 | Duygulu et al., 2011 [17] | 0 | - |
| 13 | Fitzpatrick et al., [18] | 1 | 2 |
| 14 | Hewitt et al., 2017[19] | 3 | 2 |
| 15 | Humphreys et al., 2017 [20] | 0 | - |
| 16 | Leslie et al., 2005 [21] | 1 | 3 |
| 17 | MacPhee et al., 2012 [22] | 4 | 3 |
| 18 | Miskelly et al., 2014 [23] | 1 | 2 |
| 19 | Schwarzkopf et al., 2012 [24] | 0 | - |
| 20 | Umble et al., 2011 [25] | 0 | - |
| 21 | Werrett et al., 2002 [26] | 0 | - |
| 22 | Wolbers et al., 2021 [27] | 0 | - |

# Appendix 3: Context-Mechanism-Outcome-Configurations Extracted from Included Papers on Leadership and Career Advancement Interventions Among Women in Healthcare

| Author | Setting and Participants | Brief Description of Programme | CMO-Configurations Extracted |
| --- | --- | --- | --- |
| Brown-DeVeaux et al., 2021  [6] | The mentorship diversity initiative was launched at 2 New York University (NYU) Langone Health campuses with the guidance and sponsorship of the system chief nursing officer (CNO).  NYU Langone Health is a large integrated academic medical center committed to a trifold mission to teach, serve, and discover (USA).  Participants included mentees who were nurses of a racial or ethnic minority, and mentors who were nurses working in senior leadership from multiracial backgrounds.  The intervention was directed at both genders but 94% of participants were female. | The programme consisted of 4 monthly 4-hour educational and group mentoring workshops: 1) self-efficacy and self-confidence; 2) negotiation, collaboration, and networking; 3) the paradigms of leadership; and 4) quality of life and success, as well as one-to-one mentoring. | In a large integrated academic medical centre (C) a leadership programme had buy in from executive and middle management team, aligned with the organizational goal, and comprised of education and mentorship which targeted ethnic minority participants and supported them to envision themselves in the “professional leadership framework” focused on their personal attributes via leadership education and mentoring (M- R1). The programme resulted in in ethnic minority participants increased self-efficacy to lead their team and readiness & desire for a leadership role (O). This was because ethnic minorities felt empowered given that they had received leadership training and also felt that they had the endorsement of their organizational leadership and line managers (M- R2). |
|  |  |  | In a large integrated academic medical centre (C) a mentorship programme was delivered which was part of a wider leadership programme where ethnic minority mentees aspired to advance up the career ladder and mentors were members of management and represented multiracial backgrounds who acted as role models and advisors for ethnic minority mentees who had been prepared for this via a mentorship workshop (M-R1). The programme led to an increase in participants' self-efficacy to lead their team along with readiness and desire for a leadership role (O), because ethnic minorities felt empowered due to the mentorship training thus understood how to effectively participate, and also felt they had the support of their organizational leadership and line managers (M- R2). |
|  |  |  | In a large integrated academic medical centre (C) a structured leadership education workshop which was part of a wider leadership programme was led by an expert who taught various leadership topics to ethnic minority participants who aspired to advance up the career ladder (M-R1). The programme led to an increase in participants' self-efficacy to lead their team along with readiness and desire for a leadership role (O), because ethnic minority participants felt the leadership education was relevant as the speaker highlighted the challenges the cohort had to combat when ascending the career ladder (M-R2). |
| Kelly et al., 2021 [8] | The Women’s Wellness through Equity and Leadership (WEL) programme was conceived as a collaborative effort between 6 partner organizations for physicians in the USA; the American Academy of Pediatrics (AAP), American Academy of Family Physicians (AAFP), American College of Obstetricians and Gynecologists (ACOG), American College of Physicians (ACP), American Hospital Association (AHA), and American Psychiatric Association (APA).  The programme targeted women physicians. | An 18-month curriculum was developed, which included 3 educational series on wellness, equity, and leadership. The curriculum was delivered via monthly webinars and 4 in-person meetings.  Other key components incorporated into the programme were peer support networks, diversity of programme participants and faculty, and synergy between programme education components. | The intervention programme was an initiative that originated within a network of partner organizations (C) to build leadership infrastructure among women with leadership potential by delivering a multitopic education curriculum (areas of wellness, equity and leadership) to a diverse group of women physicians which was delivered by diverse faculty using a variety of mediums and tools (in-person & online platforms, peer support, statement of impact and purpose) by taking both an individual and organizational focus (M-R1). The programme led to increased knowledge and leadership participation among women physicians (O) because women were adequately supported to succeed, and their workplace environment accommodated and encouraged their leadership (M-R2). |
|  |  |  | The intervention programme was an initiative that originated within a network of partner organizations (C) to build leadership infrastructure among women with leadership potential using a multicomponent leadership intervention, absence of mentoring was highlighted as a programme shortcoming by women physician participants (M-R1) which resulted in decreased capacity to navigate opportunities (O) because women felt inhibited when navigating new opportunities due to lack of guidance (M-R2). |
|  |  |  | The intervention programme was an initiative that originated within a network of partner organizations (C) to build leadership infrastructure among women with leadership potential by delivering a high quality multitopic education curriculum (areas of wellness, equity and leadership) to a diverse group of women physicians via in-person & online platforms during protected time so that they could focus on learning (M-R1). This led to increased knowledge and leadership participation among women physicians (O) because women believed that the education was relevant to them, they felt encouraged by meeting other participants and had the time to engage in learning (M-R2). |
| Bradd et al., 2018 [9] | Research was conducted at the Southeastern Sydney Local Health District (SESLHD) which is a large publicly funded healthcare organization, in New South Wales, Australia.  This research involved volunteer representatives from nine allied health disciplines: dietetics, occupational therapy, orthoptics, pharmacy, physiotherapy, podiatry, psychology,  social work and speech pathology.  >90% of participants in the intervention and control groups were female | The Allied Health Leadership Development Programme was conducted over a ten-month period in 2014–2015 and included three all-day workshop sessions followed by five action learning sets (ALSs). The purpose of ALS was to meet regularly with others to explore solutions to real problems and decide on the actions they wished to take. For half of the participants in the intervention (n=8), individual coaching support was also provided.  The first one-day workshop introduced leadership theory and practice development.  The second workshop comprised two full days and focused on the practical development of leadership and facilitation skills.  This session also further expounded practice development tools and methods. | A leadership initiative was developed implicitly within a government public health organization (C) for allied health professions who wanted to progress their careers vertically and the in-house leadership programme utilized existing resources and was developed and delivered by a current employee. This led to increased leadership knowledge, enhanced leadership skills and acquisition of leadership positions among participants (O) because obstacles related to delivering the programme we circumvented due to the bespoke nature of the intervention (M-R1). |
|  |  |  | A leadership initiative was developed implicitly within a government public health organization (C) for allied health professions who wanted to progress their careers vertically and the leadership development programme comprised educational workshops, coupled with action learning sets (ALS) (M-R1). This led to increased leadership knowledge, enhanced leadership skills and acquisition of leadership positions (O) because the female allied health professionals were provided with opportunity to integrate their learning after the workshops via the ALS, thus developed concrete skills (M-R2). |
|  |  |  | A leadership initiative was developed implicitly within a government public health organization (C) for allied health professions who wanted to progress their careers vertically and the leadership development programme incorporated individual coaching (M-R1) and this led to increased leadership knowledge, enhanced leadership skills and acquisition of leadership positions (O) because the female allied health professionals received individualized support to attain their goals (M-R2). |
| Dyess & Sherman, 2011 [11] | The article describes a Novice Nurse Leadership Initiative (NNLI) that was implemented 5 years ago in South Florida in the USA and continues today. It is a partnership that involved nurse leaders from 13 health care organizations, a community philanthropic foundation, and a college of nursing.  The programme targeted Novice nurses transitioning from education to employment.  93% of participants were female. | The Novice Nurse Leadership Initiative (NNLI) practice education programme included 20 sessions over 10 months to help nurses develop a leadership mindset early in their career.  Partner organizations were invited to send a minimum of 2 new nurses per cohort to ensure peer support.  A liaison was selected from each organization to aid in the facilitation of the NNLI link to  the practice environments throughout the 10 months.  Each novice was assigned a nurse mentor from their organizational setting.  A major highlight of the programme was for participants to develop evidence-based interventions that addressed practice setting challenges as part of a capstone experience. | The leadership programme was an organizational initiative (C) which was designed and delivered by nurse leaders and educators with sessions incorporating both didactic content and experiential learning (which was linked to the practice environments) delivered through face-to-face learning and web-based activities, and integrated mentorship and peer support where programme participation counted towards the novice nurses workplace role (M-R1). This led to improvement in leadership competencies and confidence among female novice nurses (O) because this was a high-quality programme with multiple avenues and supports for learning, and learning was embedded in their employment role (M-R2). |
|  |  |  | The leadership programme was a wide-ranging organizational initiative (C) that delivered structured mentoring as part of a leadership programme where novices worked with mentors and organisational leaders to identify and seek evidence based solutions to an organisational problem (M-R1) and this resulted in more global and systems perspective of nursing, enhanced leadership competencies and confidence (O) because engaging with a mentor and nurse leader to solve an organizational problem helped female novice nurses graduates make sense of what they see in practice and inspired them to take ownership as a leader in the practice environment (M-R2) |
|  |  |  | The leadership programme was a wide-ranging organizational initiative (C) and the leadership education was a subcomponent of the leadership programme, was directly linked to the practice setting, included both didactic content and experiential learning, a mix of web-based and in person activities, and was presented by nursing expert leaders (M-R1). This led to improvement in leadership competencies and improved confidence among female novice nurses (O) because the leadership education was relevant and inspired engagement as it was directly linked to the practice setting (M-R2). |
| Leggat et al., 2015 [12] | The mentoring programme was an initiative from the Australian State Department of Health.  The mentees comprised nurse practitioner candidates (NPCs) and senior nurses participated as mentors to the NPCs.  80% of participants were female. | The programme adopted an action learning approach that included elements of coaching and action learning in addition to mentoring.  Three face-to-face workshops were run for all mentors and mentees, encompassing training in mentoring skills and action learning. Educational sessions on clinical leadership were provided and there were also opportunities for reflection and feedback.  Monthly face-to-face or telephone mentoring was conducted along with monthly email contact with the programme facilitators who asked for feedback regarding progress of mentoring. | State funded public health services (C) implemented a mentoring programme where experienced, senior nurses who were not line managers of Nurse Practitioner Candidates (NPC) worked with them, to identify leadership competency gaps and develop competence (M-R1). This led to development of clinical leadership skills and transformational leadership (O) because the support and presence of role models empowered female nurse practitioner candidates (M-R2). |
|  |  |  | State funded public health services (C ) implemented a mentoring programme where leadership development was founded on action learning so that NPCs identified and developed leadership competencies relevant to their own organisational context (M-R1) which led to development of clinical leadership skills and transformational leadership among female NPCs (O). The programme was effective because content of leadership development was relevant to the contextual setting (M-R2). |
|  |  |  | State funded public health services (C ) implemented a structured mentoring approach which was overseen by a facilitator who provided feedback on the process, and training on mentoring was incorporated (for mentors and mentees) (M-R1) which led to development of clinical leadership skills and transformational leadership (O) because female NPCs and mentors were aware of how to effectively carry out the mentoring and had a facilitator to turn to if they were uncertain (M-R2). |
| Vatan & Temel, 2016 [13] | The study was conducted in 2009 at a university hospital located in İzmir, Turkey.  The nurse managers in the units were designated as mentors, and the novice nurses were protégés.  100% of participants were female. | The mentoring programme included two 4-hour training sessions to foster the nurses’ understanding about the formal process of giving and receiving mentoring.  The programme began with mentors and protégés meeting one another and discussing the best means to establish and maintain the mentoring relationship.  During the 6-month monitoring period, the researcher visited the clinics every 2 weeks to collect the reflection forms and listen to their personal experiences of the process. | University hospital clinics (C) implemented a mentorship research programme with dyads of mentor and mentee nurses from the same unit and comprised of education about mentoring for both mentors and mentees, which was followed by a formalized mentorship between experts and less experienced nurses (M-R1). This contributed to overall success of the programme as evidenced by positive change in transformational leadership for both groups, relational job learning for mentors and skill development for female mentees (O). These positive outcomes were because mentors and mentees were empowered with knowledge on how to effectively engage with the programme, and these interpersonal relationships were endorsed (M-R2). |
|  |  |  | University hospital clinics (C) implemented a mentorship research programme with dyads of mentor and mentee nurses from the same unit, comprised of mentorship which was overseen by a facilitator researcher who monitored the progress of the programme and provided clarity to mentors and mentees (M-R1) which contributed to overall success of the programme as evidenced by positive change in transformational leadership and relational job learning for mentors and female mentees (O). This was because mentors and mentees benefited from the accountability, which maintained their motivation, and they also had any issues they were facing with regards to the project resolved (M-R2). |
| Cleary et al., 2005 [16] | The programme was run at hospital and community Australian Mental Health Services.  The programme was delivered to nurses.  50% of participants were female. | This programme provided more tailored, flexible and accessible learning format utilising a purpose-designed workbook as a framework for participants to develop leadership skills.  Transformational and transactional leadership models provided the foundation for the development of the workbook resulting in a focus on individual needs, goal setting skills, self-monitoring, and regular feedback.  The role of the programme coordinators was to help identify an area of interest, assist with the development of the respondents’ learning plan, provide guidance on accessing necessary resources, and advise on the types of evidence to be submitted to demonstrate the achievement of learning aims.  An option also was given to work with other people enrolled in the clinical leadership programme who had similar interests. | An organizational initiative within mental health services (hospital and community) (C) was a flexible, self-tailored and self-directed leadership intervention delivered to mental health nurses interested in leadership development (M-R1) which resulted in enhancement of leadership qualities and activities (O) because this style of learning allowed nurses opportunity to engage with the intervention at their own convenience and developed areas that were of relevance to them (M-R2). |
|  |  |  | An organizational initiative within mental health services (hospital and community) (C) comprised a leadership programme delivered to mental health nurses interested in leadership and overseen by research facilitators who provided guidance and assistance (M-R1) which led to enhancement of leadership qualities and activities (O) because obstacles related to mental health nurses effectively engaging with the research programme were overcome with assistance of the research facilitator (M-R2). |
| Fitzpatrick et al., 2016 [18] | The Leadership Education and Development (LEAD) Programme was delivered within the Cleveland Clinic Health System (CCHS) consists of a quaternary care center with 1400 beds, 9 regional hospitals, and hospitals located in Florida, and Abu Dhabi.  The programme was delivered to nurses.  85% of participants were female. | The LEAD Programme consisted of six 4-hour sessions that took place for 3 months. The programme was focused on developing skills to empower nurses as clinical leaders and included the key concepts of personal awareness, personal leadership skills and abilities, developing communication skills, leading change, leading individuals and teams, leading others to enhance patient and provider experiences, and leadership roles for charge nurses in outcomes management. | A health system including hospitals and family health centers (C ) implemented a leadership development programme that utilised professional biographies where female nurses detailed their training, experiences, successes, and aspirations and managers were aware of the of the research and endorsed participation by selecting candidates based on demonstrated interest and leadership acumen (M-R1). The programme led to career advancement among nurses, as evidenced by applying for and attainment of new roles (O) because professional biographies served as a catalyst for nurses, prompting the realization that they had both breadth and depth in their professional experiences to attain the career positions they wanted (M-R2).The endorsement by management inspired a level of self-efficacy pertaining to their leadership. |
| Hewitt et al., 2017 [19] | The Leadership Academy (LA) is a leadership development initiative of the Society of Counselling Psychology (SCP) in America.  The programme targeted early career psychologists.  85% of participants were female. | The LA training programme spans 1 year and is facilitated by several members, referred to as the LA faculty who have served in a leadership capacity.  The programme commences with an assessment of participants interpersonal needs, and these results are integrated into the curriculum.  The participant works throughout the year to design and implement a service project with the assistance of a mentor. The purpose of this project is to assist LA participants in recognizing and developing their leadership potential. Participants are responsible for selecting their own mentor. | As an initiative of the Society of Counselling Psychology (C) a leadership development programme was implemented where psychologists designed and implemented a service project with the support of a mentor who shared aspects of the psychologist’s social identity and demonstrated leadership in various scenarios where mentees could observe and learn (M-R1). The programme resulted in developing leadership skills and confidence to lead among female psychologists (O) because this built the self-efficacy of the psychologists who shared a social identity with the mentors (M-R2). |
|  |  |  | As an initiative of the Society of Counselling Psychology (C) a leadership development programme was implemented where psychologists design and implement a service project but are initially assessed for interpersonal skills and needs as this relates to leadership (M-R1). The programme led to the development of leadership skills and confidence to lead among female psychologists (O) because the psychologists had a greater understanding and appreciation for their personal leadership styles and consequently understood how to further develop (M-R2). |
|  |  |  | As an initiative of the Society of Counselling Psychology (C) leadership development programme was implemented where psychologists design and implement a service project and the programme  incorporates learning on the organizations and leadership positions (M-R1) which led to increased confidence in leadership and greater understanding of organizational structures(O) because knowledge on the organization and leadership positions aided the psychologists in gauging how aligned they were with the requirements of the positions (M-R2). |
| Leslie et al., 2005 [21] | The Johnson & Johnson Pediatric Institute, the American Academy of Pediatrics developed a 1-year strategy to train pediatricians who are <40 years old or <5 years in practice in leadership skills in the USA.  55% of participants were female. | The 3-day training programme began with an overview of the meeting’s philosophy, agenda, and leaders followed by 2 full 14-hour days of didactic and small-group programmes centred on the core areas of curriculum.  The programme combined didactic sessions with other modalities, shown to enhance participant interaction and provide creative opportunities to practice skills.  These other modalities included role-playing, team projects, development of brief “infomercials,” small-group discussions and reporting out, self-assessment surveys, case studies, review of videotaped scenarios, hands-on workshops, and behavioural change contracts (a form of goal setting). | As a strategy from the American Academy of Paediatrics to train new paediatricians (C) a leadership development programme was run for paediatricians nominated by the organisation and included didactic sessions and interactive sessions (role playing, team projects, group discussions), and also incorporated a learning contract where participants set leadership goals and reported on progress of this contract (M-R1). This programme led to achievement of the leadership oriented goals (O ) because the learning contract reinforced lessons learnt from the programme and provided accountability (M-R2). |
| MacPhee et al., 2012 [22] | This provincially funded programme was specifically designed for novice front-line nursing leaders in Canada.  89% of participants were female. | The Nursing Leadership Institute (NLI) is a year-long programme with four components: (i) a 4-day workshop; (ii) mentoring support from higher level leadership; (iii) organizational supports to implement leadership projects in the practice environment; and (iv) virtual networking (i.e. online community of practice). | A state initiative was carried out and (C) comprised a leadership development programme for front-line nurse leaders where they were connected with mentors from their workplace who could support them with programme-related leadership development (M-R1) which resulted in the female nurses fulfilling their leadership roles (O) because the front-line nurses were able to overcome obstacles related to satisfying the programme requirements due to the mentor’s support (M-R2). |
|  |  |  | A state initiative was carried out and (C) comprised a leadership development programme for front-line nurse leaders where they were provided with resources and tools (e.g. communication) (M-R1) which resulted in the female nurses utilising the tools in fulfilment of leadership roles (O) because these tools made leadership easier or more efficient (M-R2). |
|  |  |  | A state initiative was carried out and (C) comprised a leadership development programme for front-line nurse leaders where the training was tailored to each person’s leadership style (M-R1) which resulted in confidence in own leadership style (O) because the female nurse leaders felt that they each had something unique to offer and thus felt motivated (M-R2). |
|  |  |  | A state initiative was carried out and (C) comprised a leadership development programme for front-line nurse leaders that provided opportunity for networking with those of similar profession (M-R1) which led to effective fulfilment of leadership roles among the female nurses (O) because the networking provided opportunities to learn and obtain reassurance (M-R2). |
| Miskelly & Duncan, 2014 [23] | An in-house nursing and midwifery leadership programme within the New Zealand District Health Board for nurses and midwives.  97% of participants were female. | Pebbles was run over a 6-month period, with participants released from clinical duties for a full day once a month for 6 months.  Facilitation of the programme was carried out by coordinators practice development for nurses and comprised of elements including group discussions, Case studies, visiting the library, reviewing and critiquing academic literature, as well as presentations by senior nurses. Participants completed assignments  between each session and presented an aspect of their clinical practice to the group. | A New Zealand District Health Board initiative (C ) run a leadership development programme for nurses and midwives comprising primarily of education where participants were nominated by their managers (M-R1). This resulted in increased confidence to lead among female nurses and midwives (O) because their leaders believed in them (M-R2). |

# References

1. Mousa M, Boyle J, Skouteris H, Mullins AK, Currie G, Riach K, Teede HJ: **Advancing women in healthcare leadership: A systematic review and meta-synthesis of multi-sector evidence on organisational interventions**. *EClinicalMedicine* 2021, **39**:101084.

2. House A, Dracup N, Burkinshaw P, Ward V, Bryant LD: **Mentoring as an intervention to promote gender equality in academic medicine: a systematic review**. *BMJ Open* 2021, **11**(1):e040355.

3. Laver KE, Prichard IJ, Cations M, Osenk I, Govin K, Coveney JD: **A systematic review of interventions to support the careers of women in academic medicine and other disciplines**. *BMJ Open* 2018, **8**(3):e020380.

4. Alwazzan L, Al-Angari SS: **Women’s leadership in academic medicine: a systematic review of extent, condition and interventions**. *BMJ Open* 2020, **10**(1):e032232.

5. Lydon S, Dowd E, Walsh C, Dea A, Byrne D, Murphy AW, Connor P: **Systematic review of interventions to improve gender equity in graduate medicine**. *Postgraduate Medical Journal* 2022, **98**(1158):300.

6. Brown-DeVeaux D, Jean-Louis K, Glassman K, Kunisch J: **Using a Mentorship Approach to Address the Underrepresentation of Ethnic Minorities in Senior Nursing Leadership**. *The Journal of nursing administration* 2021, **51**(3):149-155.

7. Chang A, Lundebjerg NE, Abrams J, Barnes DE, Fain MJ, Hall WJ, Johnson TM, Michael Harper G, Williams B, Ritchie CS: **Leadership, Inside and Out: The Tideswell‐AGS‐ADGAP Emerging Leaders in Aging Program**. *Journal of the American Geriatrics Society (JAGS)* 2019, **67**(3):437-442.

8. Kelly EH, Miskimen T, Rivera F, Peterson LE, Hingle ST: **Women’s wellness through equity and leadership (WEL): A program evaluation**. *Pediatrics (Evanston)* 2021, **148**(Suppl 2):1.

9. Bradd P, Travaglia J, Hayen A: **Developing allied health leaders to enhance person-centred healthcare**. *Journal of Health Organization and Management* 2018, **32**(7):908-932.

10. Spiva L, Davis S, Case-Wirth J, Hedenstrom L, Hogue V, Box M, Berrier E, Jones C, Thurman S, Knotts K *et al*: **The Effectiveness of Charge Nurse Training on Leadership Style and Resiliency**. *The Journal of nursing administration* 2020, **50**(2):95-103.

11. Dyess S, Sherman R: **Developing the leadership skills of new graduates to influence practice environments: a novice nurse leadership program**. *Nurs Adm Q* 2011, **35**(4):313-322.

12. Leggat SG, Balding C, Schiftan D: **Developing clinical leaders: the impact of an action learning mentoring programme for advanced practice nurses**. *Journal of clinical nursing* 2015, **24**(11-12):1576-1584.

13. Vatan F, Temel AB: **A leadership development program through mentorship for clinical nurses in Turkey**. *Nursing economic* 2016, **34**(5):242-250.

14. Al-Hussami M, Hamad S, Darawad M, Maharmeh M: **The effects of leadership competencies and quality of work on the perceived readiness for organizational change among nurse managers**. *International journal of health care quality assurance incorporating Leadership in health services* 2017, **30**(4):443-456.

15. Clark TJ, Yoder-Wise PS: **Enhancing Trifocal Leadership Practices Using Simulation in a Pediatric Charge Nurse Orientation Program**. *The Journal of continuing education in nursing* 2015, **46**(7):311-317.

16. Cleary M, Freeman A, Sharrock L: **THE DEVELOPMENT, IMPLEMENTATION, AND EVALUATION OF A CLINICAL LEADERSHIP PROGRAM FOR MENTAL HEALTH NURSES**. *Issues in mental health nursing* 2005, **26**(8):827-842.

17. Duygulu S, Kublay G: **Transformational leadership training programme for charge nurses: Transformational leadership training programme**. *Journal of advanced nursing* 2011, **67**(3):633-642.

18. Fitzpatrick JJ, Modic MB, Van Dyk J, Hancock KK: **A Leadership Education and Development Program for Clinical Nurses**. *The Journal of nursing administration* 2016, **46**(11):561-565.

19. Hewitt AA, Watson LB, DeBlaere C, Dispenza F, Guzmán CE, Cadenas G, Tran AGTT, Chain J, Ferdinand L: **Leadership Development in Counseling Psychology: Voices of Leadership Academy Alumni**. *The Counseling psychologist* 2017, **45**(7):992-1016.

20. Humphreys BP, Kurtz AJ, Portrie C, Couse LJ, Hajnaghizadeh F: **Advancing Leadership Skills: A Multiyear Examination of LEND Trainee Self-Efficacy Growth**. *Maternal and child health journal* 2018, **22**(10):1377-1383.

21. Leslie LK, Miotto MB, Liu GC, Ziemnik S, Cabrera AG, Calma S, Huang C, Slaw K: **Training Young Pediatricians as Leaders for the 21st Century**. *Pediatrics (Evanston)* 2005, **115**(3):765-773.

22. MacPhee M, Skelton-Green J, Bouthillette F, Suryaprakash N: **An empowerment framework for nursing leadership development: supporting evidence: Empowerment framework for nursing leadership development**. *Journal of advanced nursing* 2012, **68**(1):159-169.

23. Miskelly P, Duncan L: **'I'm actually being the grown-up now': leadership, maturity and professional identity development**. *Journal of Nursing Management* 2014, **22**(1):38-48.

24. Schwarzkopf R, Sherman RO, Kiger AJ: **Taking Charge: Front-Line Nurse Leadership Development**. *The Journal of continuing education in nursing* 2012, **43**(4):154-159.

25. Umble KE, Baker EL, Woltring C: **An Evaluation of the National Public Health Leadership Institute—1991-2006: Part I. Developing Individual Leaders**. *Journal of public health management and practice* 2011, **17**(3):202-213.

26. Werrett J, Griffiths M, Clifford C: **A regional evaluation of the impact of the Leading an Empowered Organisation leadership programme**. *NT research* 2002, **7**(6):459-470.

27. Wolbers I, Lalleman PCB, Schoonhoven L, Bleijenberg N: **The Ambassador Project: Evaluating a Five-Year Nationwide Leadership Program to Bridge the gap Between Policy and District Nursing Practice**. *Policy, politics & nursing practice* 2021, **22**(4):264-275.
